# Irregular Action of the Heart

**Published:** 1913-03-08

**Authors:** 


### IRREGULAR ACTION OF THE HEART.

### The New Views, Terminology, and Instruments.

Any sister-in-charge of a medical ward would bear witness to the increasing complexity of the various instruments by which her visiting physicians and the house physicians and clerks apply themselves to the study of cases of heart disease. With the advent of these often wonderful and fearful machines—sphygmo-manometers, polygraphs, galvanometers, sphygmographs, and what not—a new and often apparently involved nomenclature has come into use. Whereas ten years ago the stethoscope was almost the only instrument which the physician required for investigating a case of heart disease, and the four common valve lesions monopolised nine-tenths of his attention and treatment, nowadays a whole series of new terms has passed into current circulation, of which even the greatest authorities on heart disease a decade ago had never heard.

This revolution has, naturally, not been brought about suddenly, but gradually during the past few years. If there is one man more than another who can be regarded as the parent of these profound changes and advances, Dr. James Mackenzie deserves that honour. He has been the inspirer of quite a considerable school, among whom Dr. T. Lewis, of University College Hospital, has been perhaps the most notable in London. But the work of these physicians is all based on the bedrock of research in physiology; and the author of those researches is the veteran Dr. W. H. Gaskell, of Cambridge, who laid the foundations thirty years ago upon which the physicians have lately been building. There have, of course, been many others who have helped in the rise of cardiac pathology to its present level. Professor A. Waller, for instance, in this country, and Professor Einthover abroad.

The edifice thus erected, or, rather, in process of erection, is likely to endure, for its foundations have been well and truly laid. The new terminology and the new views have, in fact, come to stay, though they may be greatly modified in detail by further researches. It therefore behoves those members of the medical profession who have, as a rule, little or nothing to do with cases of heart disease, as well as those in auxiliary professions, such as that of nursing, to obtain a general outline idea of what these new ideas are. Specialists, of course, have to delve much more deeply into the matter, and general practitioners are under much the same obligation, if they are able to spare the time, which very few can. Fortunately, it is not necessary to master the technique of the ink polygraph or the string galvanometer in order to get a grip of the meaning of sinus irregularity, auricular fibrillation, and the rest of the Mackenzie nomenclature. Even the diagnosis of these conditions can often be made, or approximated to, without the use of these special instruments, by attending to some of the points which the researches of the new school have established. For a concise and methodical survey of this—the less technical aspect of the new cardiac pathology—the thanks of those who take an interest in heart cases without aspiring to be experts are due to Dr. Langdon Brown,* who explains very clearly the main kinds and varieties of irregularity of the heart as now classified by the Mackenzie school.

For our present purpose it is unnecessary to go quite as deeply as Dr. Brown does into comparative anatomy and physiology. It suffices to explain that the automatic beat of the heart is a function which originates, not in the whole muscular wall, but in a certain small portion of it just where the vena cava enters the right auricle. From this point the impulse of contraction spreads along the auricular wall until it reaches a point near the coronary sinus; here a small band of muscle conveys it from the auricle into the ventricle, and this is called the auriculo-ventricular bundle. Interference with the regular action of the heart may be due to default in any of these components of the contracting and conducting mechanism. If the output of contractile impulses from the sinus venosus is not absolutely regular, "sinus irregularity" is the result. If the muscular wall of the auricle fails to convey regularly the contractile impulse of a normal sinus venosus, "auricular fibrillation" is predicated. If the sinus and the auricle function normally, but the auriculo-ventricular bundle fails to pass on every beat, "heart block" is said to exist. Lastly, "pulsus alternans" is caused by exhaustion of the contractile power of the heart muscle.

In "sinus irregularity" the periodic contractile impulse is originated at the normal place, but not at the normal time. Each wave of contraction, once started, proceeds normally; and since the actual wave thus takes up the proper time, it is the quiescent period between the beats which varies. This type of irregularity is met with in a proportion of quite healthy people; and even in illness it has no especial significance. Curiously, in fever it disappears, reappearing sometimes when the temperature drops. Sinus irregularity, says Dr. Brown, can be recognised by the fact that, although the pulse rate is variable usually with respiration, the beats are equal in strength; while on auscultation the interval between the first and second sounds is constant.

"Auricular fibrillation," however, is practically always a sign of an organic heart lesion, not necessarily valvular disease. The pulse is irregular to a degree. The intervals and nature of the beats are entirely disorderly, and the rhythm is usually rapid. In cases of mitral stenosis, in which auricular fibrillation is especially common, the crescendo presystolic murmur disappears when this complication sets in. The veins of the neck are distended, and the pulse in them is synchronous with the ventricular systole. Subjectively, dyspnœa,

* *The Medical Review*, January 1913, p. 1.

orthopnœa, angina, and other symptoms of severe cardiac distress may be expected.

"Heart block" is the name now given to the condition known as the Stokes-Adams syndrome. The sinus and the auricle start the proper number of waves, but many of them fail to get through the ventricular bundle into the ventricle, which accordingly contracts very slowly—forty, thirty, twenty, or even fewer times a minute. Syncope and fits of various kinds are not uncommon in this condition. Anything which affects the narrow auriculo-ventricular bundle may produce heart block, but syphilis is the commonest cause of it. The condition is occasionally temporary, the bundle afterwards recovering; but usually it is permanent and progressive.

"Pulsus alternans" is less easily to be recognised without elaborate instruments. It consists of alternate stronger and weaker beats, and is seen especially in cases of high blood-pressure from arterio-sclerosis. Exercise may cause it to appear, when during rest it is not apparent. The prognosis of this kind of irregularity is extremely serious, and, therefore, it is important not to confuse it with the irregularity due to ventricular extra systoles. In the latter a long pause follows a small beat, whereas in pulsus alternans the shorter pause follows the small beat. Extra systoles, wherever they originate, are due to increased irritability of the heart; and are not necessarily a sign of serious cardiac derangement. In pulsus alternans digitalis does harm; and it is also not to be given in incomplete heart block. Indeed, one of the most important practical applications of the new cardiac pathology is in the light which has been shed upon the indications and contra-indications for digitalis, strophanthus, caffeine, and other heart stimulants. Into these points Dr. Brown's instructive article enters fully; and those who wish to follow up this aspect more fully cannot do better than consult it in the original.

